# DXA-derived hip shape is related to osteoarthritis: findings from in the MrOS cohort

**DOI:** 10.1016/j.joca.2017.09.006

**Published:** 2017-12

**Authors:** B.G. Faber, D. Baird, C.L. Gregson, J.S. Gregory, R.J. Barr, R.M. Aspden, J. Lynch, M.C. Nevitt, N.E. Lane, E. Orwoll, J.H. Tobias

**Affiliations:** † Musculoskeletal Research Unit, School of Clinical Sciences, University of Bristol, Southmead Hospital, Bristol BS10 5NB, UK; ‡ Arthritis and Musculoskeletal Medicine, Institute of Medical Sciences, University of Aberdeen, AB25 2ZD, UK; § Department of Epidemiology and Biostatistics, University of California San Francisco, California, USA; ‖ Department of Medicine, University of California Davis, Sacramento, CA, USA; ¶ Division of Endocrinology, Oregon Health & Science University, Portland, USA

**Keywords:** Hip shape, Joint shape, Active shape modelling

## Abstract

**Objective:**

Statistical shape modelling (SSM) of radiographs has been used to explore relationships between altered joint shape and hip osteoarthritis (OA). We aimed to apply SSM to Dual-energy X-ray Absorptiometry (DXA) hip scans, and examine associations between resultant hip shape modes (HSMs), radiographic hip OA (RHOA), and hip pain, in a large population based cohort.

**Method:**

SSM was performed on baseline hip DXA scans from the Osteoporotic Fractures in Men (MrOS) Study. Associations between the top ten HSMs, and prevalent RHOA from pelvic radiographs obtained 4.6 years later, were analysed in 4100 participants. RHOA was defined as Croft score ≥2. Hip pain was based on pain on walking, hip pain on examination, and Western Ontario and McMaster Universities Arthritis Index (WOMAC).

**Results:**

The five HSMs associated with RHOA showed features of either pincer- or cam-type deformities. HSM 1 (increased pincer-type deformity) was positively associated with RHOA [1.23 (1.09, 1.39)] [odds ratio (OR) and 95% CI]. HSM 8 (reduced pincer-type deformity) was inversely associated with RHOA [0.79 (0.70, 0.89)]. HSM 10 (increased cam-type deformity) was positively associated with RHOA [1.21 (1.07, 1.37)]. HSM 3 and HSM 4 (reduced cam-type deformity) were inversely associated with RHOA [0.73 (0.65, 0.83) and 0.82 (0.73, 0.93), respectively]. HSM 3 was inversely related to pain on examination [0.84 (0.76, 0.92)] and walking [0.88, (0.81, 0.95)], and to WOMAC score [0.87 (0.80, 0.93)].

**Conclusions:**

DXA-derived measures of hip shape are associated with RHOA, and to a lesser extent hip pain, possibly reflecting their role in the pathogenesis of hip OA.

## Introduction

Hip osteoarthritis (OA) is an increasingly important cause of morbidity as the mean age of the population increases^1^. Identification of underlying risk factors may open up new avenues for preventative strategies. One of the most important is abnormalities of hip development leading to alterations in hip shape, exemplified by developmental dysplasia of the hip which is screened for routinely in neonates^2^, ^3^. More subtle alterations in hip shape have also been reported to be associated with hip OA. For example, cam-type deformities, caused by extra bone growth around the anterolateral aspect of the femoral head–neck junction resulting in a non-spherical femoral head, leading to femoro-acetabular impingement (FAI)^4^, are associated with premature onset of OA^5^, ^6^. FAI may also result from a pincer-type deformity where the acetabulum overhangs and encroaches on the lateral aspect of the femoral head, for which currently there is contradictory evidence in terms of associations with OA^2^, ^5^.

In the above studies, hip shape was defined using geometric parameters measured on radiographs such as femoral neck or centre-edge angle. An alternative approach, statistical shape modelling (SSM), has been developed whereby principal component analysis (PCA) is used to derive a set of orthogonal hip shape modes (HSMs), which together provide a more complete description of hip shape^7^. Using this method, changes to the lateral curvature of the femoral head^7^, and larger femoral head relative to femoral shaft^8^ have been reported to be associated with more rapid progression of radiographic hip OA (RHOA) and, interestingly, with prevalent knee OA^9^. However, these studies were based on SSM of the femoral head alone, additional information is provided by models which also include the acetabulum^10^. For example, in a recent study by Agricola *et al.* using a combined femoral head and acetabulum SSM, a retroverted acetabulum (defined as the posterior acetabular wall located medially with respect to the centre of the femoral head) was found to be predictive of RHOA^11^.

A limitation of the above approaches towards studying hip shape is their reliance on use of radiographs. Whereas sample sizes based on radiographic collections are large enough for conventional epidemiological studies, they provide limited power for genetic studies. Lindner *et al.* examined genetic influences on hip shape in 929 cases of unilateral RHOA, observing associations between three loci and hip shape following a look up of 41 candidates^12^. However, considerably larger samples, including unaffected individuals, are required to perform genome wide association studies (GWAS) intended to identify novel genetic loci. For example, in the osteoporosis field, the largest GWAS study to date identified 56 loci associated with bone mineral density (BMD) of which 32 were novel, based on Dual-energy X-ray Absorptiometry (DXA) scans from over 90,000 individuals from population based cohorts^13^. Widely available hip DXA scans may also prove useful in evaluating relationships between hip shape and hip OA. For example, Waarsing *et al.* applied a statistical model combining shape and density from hip DXA scans in 218 patients with hip OA, following which several modes were found to be associated with features of RHOA^14^. However, to what extent pure shape measures derived from hip DXA scans are also related to RHOA, and whether similar relationships are observed in population-based cohorts, is currently unclear.

To establish whether DXA-derived hip shape represents a useful phenotype for future GWAS studies intended to identify novel genetic risk factors for hip OA, in the present study, we aimed to examine whether hip shape derived from a SSM applied to hip DXA scans is associated with RHOA in the Osteoporotic Fractures in Men (MrOS) Study; in this population-based cohort, hip radiographs were performed a mean of 4.6 years following baseline DXA scans. Given the lack of concordance between radiographic findings and symptoms in hip OA^15^, we also aimed to examine to what extent hip shape shows equivalent associations with hip pain, ascertained at the same time as hip radiographs using a combination of questionnaires and examination.

## Methods

### Study participants

The MrOS cohort, within which this cross-sectional study is based, is a prospective study of 5,994 men recruited between 2000 and 2002 at six centres around the United States (Birmingham, Alabama; Minneapolis, Minnesota; Palo Alto, California; the Monongahela Valley near Pittsburgh, Pennsylvania; Portland, Oregon; and San Diego, California). To be eligible, men had to be ≥65 years old, ambulatory, and without bilateral hip replacements. A full description of the MrOS cohort has been previously published^16^, ^17^. We used hip shape data derived from DXA scans performed at the baseline visit, as part of a separate study examining genetic influences on this phenotype. Pelvic X-rays for assessing RHOA, and hip examination and symptoms questionnaire, were obtained as part of a second visit conducted from March 2005 to May 2006, on average 4.6 years later.

### Demographic characteristics

All demographic information is taken from visit one. The participant's age was taken as the age in years at their last birthday. A Harpenden stadiometer (Holtain Ltd, Crymych, Wales) measured standing height in centimetres, which was based on an average of two readings, if these differed by ≥4 mm, two further readings were taken. Weight was measured to the nearest 0.1 kg using a standard balance-beam scale or digital scales using standard protocols. Race was a self-identified criterion with the participants asked to select one of the following: white, African American, Asian, native Hawaiian or other Pacific Islander, American Indian or Alaskan native, multi-racial and unknown.

### DXA protocol

Right hip DXA imaging was performed at the baseline visit unless they had a right hip replacement in which case a left hip scan was performed. A QDR 4500 Hologic machine (Waltham, MA) was used at all six sites. There was a standardized protocol for positioning participants and all DXA technicians were certified centrally^18^.

### SSM

**Fig. 1:**
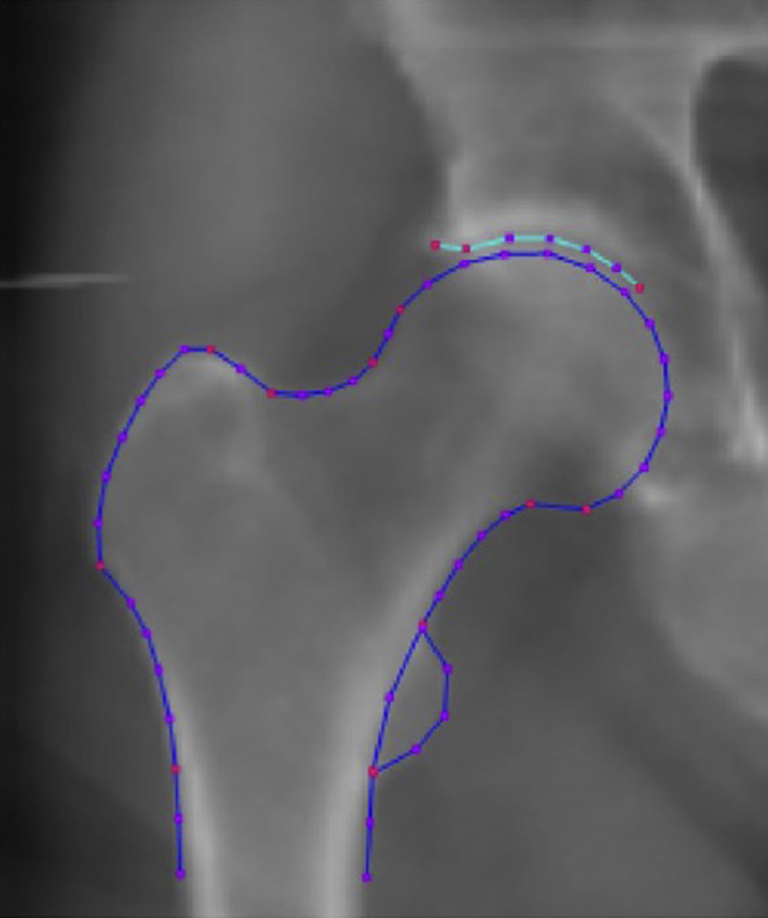
An example DXA image. This is a DXA image taken from the MrOS cohort. The 58 points used for the SSM are marked on the image. Key points are marked in red and these represent anatomical reference points to ensure accurate marking. There are two key lateral acetabular points placed on the outer edge of the acetabulum and one key medial acetabular point placed at the end of the acetabular eyebrow. The other key points are located around the trochanters, the femoral head and the femoral neck.

Hip DXA scans were uploaded to SHAPE software (University of Aberdeen). A 58-point model was used that automatically placed points around anatomical landmarks of the upper femur and adjacent acetabulum; all images were reviewed and, where necessary, points were manually re-positioned by a trained operator to ensure they were positioned on the bone edge (Fig. 1). Before marking up MrOS scans, a training set of 100 images was used to ensure accurate point placement. Median point-to-point difference (i.e., distance between a given point placed by the operator and the average point after combining placements by all operators) was derived for each operator, a score of ≤3 pixels denoting acceptable accuracy. Firstly, Procrustes analysis was performed to transform the points without deformation by scaling, rotation and translation so that they are aligned as closely as possible, this is followed by PCA. SHAPE is based on the algorithm first validated by Cootes *et al.* when measuring the shape of resistors, heart chambers and hands and more recently validated by Linder *et al.* against manually derived geometric measures from hip radiographs^19^, ^20^. SHAPE through SSM produces linearly independent variations in hip shape (HSM)^21^, ^22^. Each mode was normalized to zero mean and unit standard deviation (SD) for the whole cohort so that each image (and therefore participant) is assigned a set of mode scores in units of SDs describing how far they lie from the mean. Images producing HSM scores above or below 4SDs were manually checked by two operators, and point placement corrected where necessary. Mode shapes were subsequently assigned to cam or pincer-type deformities based on consensus visual interpretation.

### RHOA

At visit two, standing pelvic radiographs were performed using a standardized protocol. Each radiograph was read by a primary reader and scored, using a previously published atlas^23^, for concentric, medial or lateral joint space narrowing (JSN) (0–4), osteophyte formation at the inferior and superior femur and acetabulum (0–3), bone cysts (0–3), subchondral sclerosis (0–3), and femoral head deformity (0–3)^18^. In addition, binary measures were created for the presence of osteophytes, JSN or subchondral sclerosis, based on mild (grade ≥1) used for primary analyses, moderate (grade ≥2) or severe (grade ≥3) cut-offs. Radiographs with definite osteophytes or JSN were then examined by a second reader to reach a consensus score. Croft scores, based on an aggregate of these scores, were subsequently generated^24^, with a score ≥2 (requiring the presence of osteophytes or JSN) taken as the presence of moderate RHOA which was used in primary analyses, and a score ≥3 denoting severe RHOA^18^.

### Hip pain

All participants who attended visit two were asked to undergo a hip examination. The participant's right hip was internally rotated and patient-reported pain was documented generating a binary outcome. Participants completed a questionnaire concerning right hip pain on walking in the last 30 days (scored 0–4), which was converted to a binary measure i.e., presence or absence of right hip pain on walking in the last 30 days. Finally, all patients had a standardized Western Ontario and McMaster Universities Arthritis Index (WOMAC) score calculated out of 20. The WOMAC score, which has been validated as a measure of hip OA^25^, encompasses pain, stiffness and function to give an overall score of disease, 0 being no limitation and 20 being severe limitation.

### Statistical analysis

To limit multiple testing, we restricted our analyses to the ten HSMs explaining the greatest proportion of variance in hip shape. Demographic statistics were summarized as mean (SD) for continuous variables and counts (percentages) for categorical variables. Logistic regression was used to analyse associations between each of these HSMs as separate predictors, modelled as continuous variables, and binary OA outcomes; ordinal logistic regression was used to examine relationships with WOMAC pain score outcomes, results are given as an odds ratio (OR). Sensitivity analyses were also performed where we compared the results after applying different Croft score definitions for OA, and different cut-offs for defining osteophytes. In the adjusted regression models we adjusted for age, height, weight and race as *a priori* confounders, as recorded at visit one. In setting *P* values for the strength of evidence against the null hypothesis, we considered our top ten HSMs as independent exposures, and a global Croft score of ≥2 (indicating at least moderate OA) as our primary outcome, based on our fully adjusted model, giving a Bonferroni-corrected *P* value of 0.005. All statistical analysis used Stata release 14 statistical software (StataCorp, College Station, TX, USA).

## Results

### Population characteristics

Of the 5994 MrOS participants attending visit one, right DXA scans were available in 5862 (97.8%), having excluded those with incomplete data (*N* = 86), previous joint replacement (*N* = 45) or poor image quality (*N* = 1), from which hip shape was generated. At this baseline visit, participants were a mean of 72.8 years of age, 83.6 kg in weight, and 174.4 cm in height, giving mean Body Mass Index (BMI) of 27.5 kg/m^2^. At visit 2 (a mean of 4.6 years later), right hip radiographs were read for RHOA, which were available for 4100 (69.9%) of these participants, who formed the basis of the present study, of whom 90.7% were white, 3.3% Asian, 3.2% African American and 2.8% multiracial/unknown/other.

At visit 2, 7.1% had evidence of RHOA, based on Croft score ≥2 (Table I). Lateral acetabular osteophytes were the most common radiographic feature of hip OA, with any osteophyte at this site present in 19.2% of participants. Furthermore, at visit 2, 11.4% had hip pain on examination, and 20.2% reported hip pain on walking.

**Table I:** Prevalence of radiographic and hip OA and hip pain

	Prevalence *n* [%]
**Radiographic OA**	
Croft < 2	3811 [93]
Croft ≥ 2	289 [7.1]
Croft ≥ 3	100 [2.4]
*Any osteophyte (i.e., score* *≥* *1)*	
Lateral acetabular	788 [19.2]
Lateral femoral	401 [9.8]
Inferior acetabular	404 [9.9]
Inferior femoral	272 [6.6]
*Any joint space narrowing (i.e., score* *≥* *1)*	
Lateral	207 [5.1]
Medial	446 [10.9]
Concentric	148 [3.6]
*Other bone lesions*	
Cysts	44 [1.1]
Any subchondral sclerosis (i.e., score ≥ 1)	278 [6.8]
Chondrocalcinosis	9 [0.02]
Joint deformity	35 [0.9]
**Symptoms**	
Hip pain on examination	451 [11.4]
Hip pain on walking	829 [20.2]
WOMAC	0.9 [2.3, 0, 20.0]

Prevalence based on 4100 individuals with right hip X-rays. Results are shown as prevalence [%], apart from the WOMAC score which is presented as mean [SD, Min, Max]. *N* = 4,100 except for pain on examination (*N* = 3,946), walking (*N* = 4,098) and WOMAC score (*N* = 4,076).

### HSMs

**Fig. 2:**
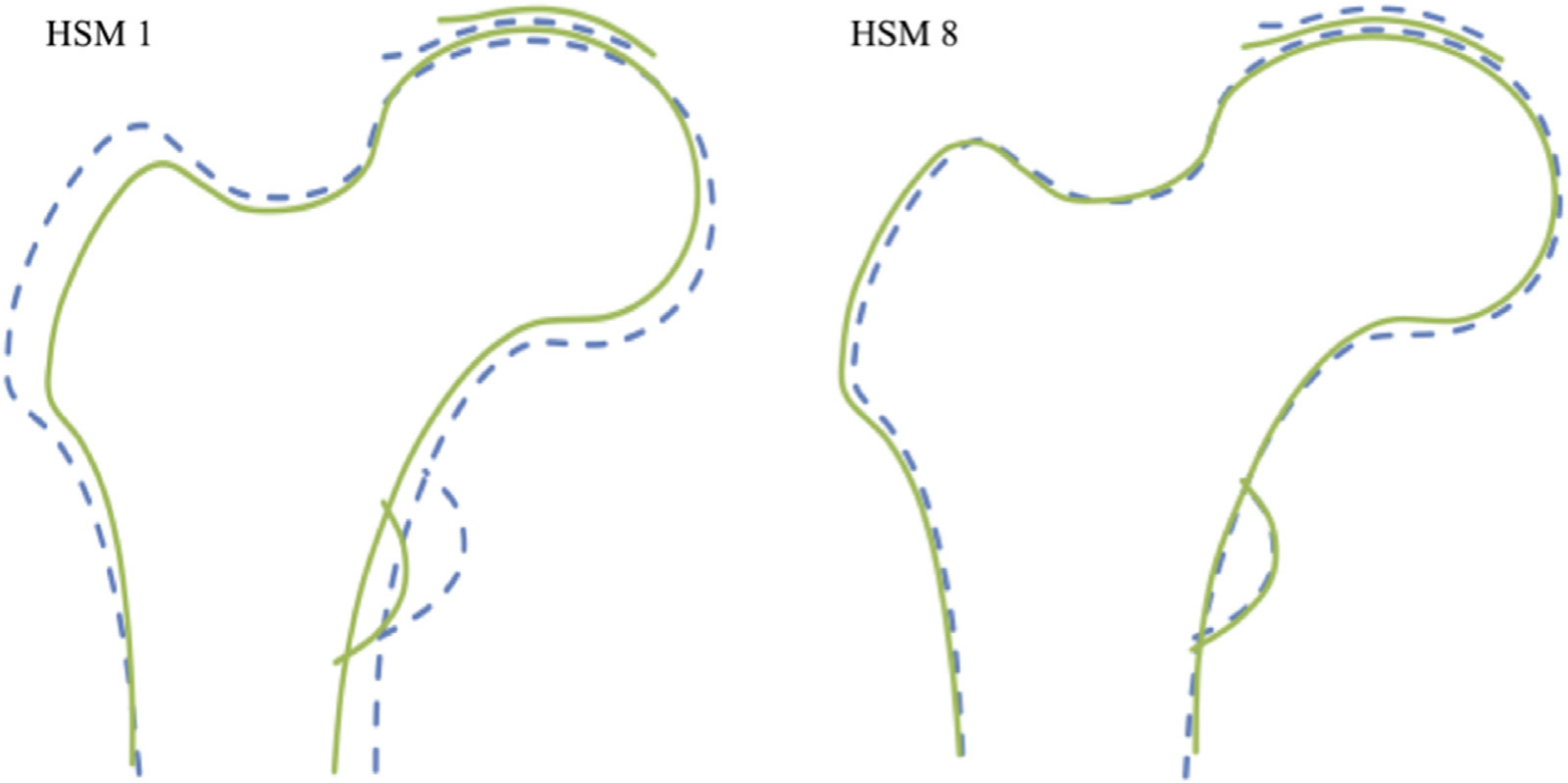
HSMs 1 and 8. Pincer-type variations in hip shape demonstrated by HSM 1 and HSM 8. HSM 1 has a positive relationship with a pincer-type variation. HSM 8 has a negative relationship with a pincer-type variation. Dashed line = +2 SDs, solid line = −2SDs.

**Fig. 3:**
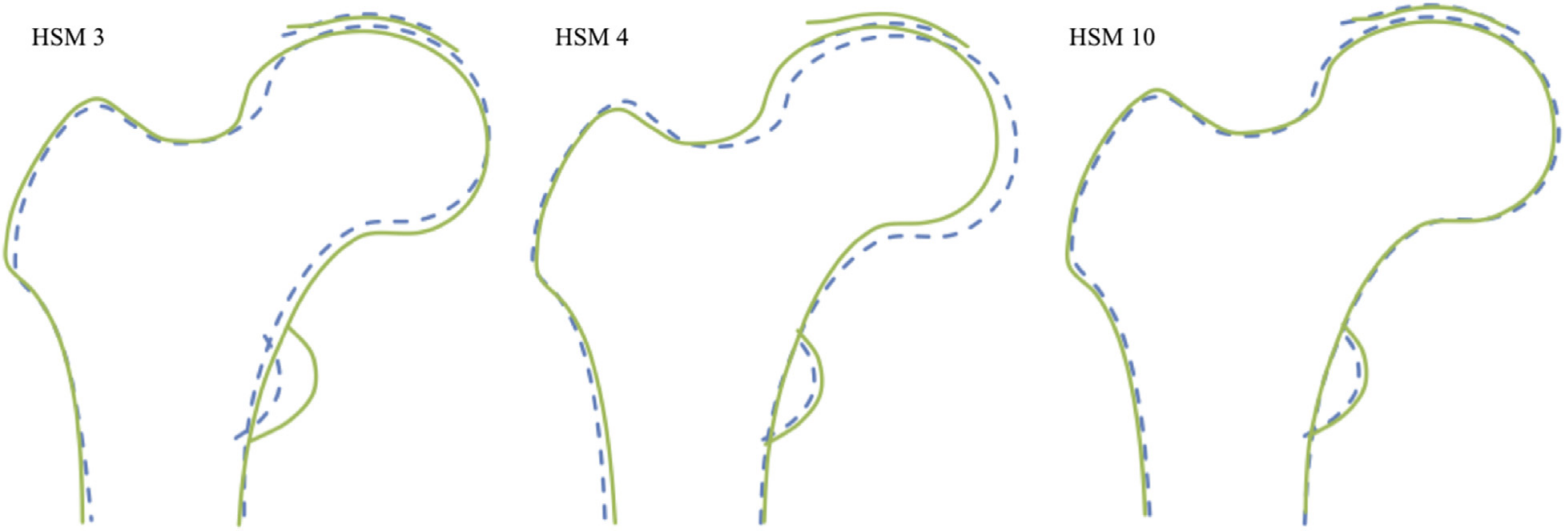
HSMs 3, 4 and 10. Cam-type variation in hip shape demonstrated by HSMs 3, 4 and 10. HSMs 3 and 4 have a negative relationship with a cam-type variation. HSM 10 has a positive relationship with a cam-type variation. Dashed line = +2 SDs, solid line = −2SDs.

The first ten HSMs in our cohort explained 81.4% of the total variance in hip shape. Five HSMs were found to be associated with RHOA (see below), which together explained 48.3% of the total variance in hip shape. All five HSMs associated with RHOA showed features of FAI on visual inspection, either in the form of pincer- or cam-type deformities (Fig. 2, Fig. 3), whereas the remaining HSMs were unrelated to these deformities. No HSM was related to both deformities, implying these represent statistically independent contributions to hip shape. HSM 1, which accounted for 22.3% of total variance in hip shape, was positively associated with pincer-type deformity (Fig. 2). HSM 8, which accounted for 2.8% of total variance in hip shape, was negatively associated with pincer-type deformity. HSM 3 and HSM 4, which explained 12.1% and 9.2% of total variation in hip shape, respectively, were negatively associated with cam-type deformity (Fig. 3). HSM 10, which explained 1.9% of total variation in hip shape, was positively associated with cam-type deformity.

These modes also reflected other shape differences. HSM 1 was associated with a larger femoral head, larger lesser and greater trochanters, wider femoral neck width, and narrower supero-medial joint space; HSM 3 was related to a smaller lesser trochanter and narrower supero-medial joint space; HSM 4 was associated with altered neck shaft angle leading to medial displacement of the femoral head; and HSM 8 was related to a wider supero-lateral joint space. In contrast, the HSMs not associated with measures of hip OA were unrelated to pincer- or cam-type deformities; HSM 2 featured a smaller femoral head with a steeper femoral neck angle and larger lesser trochanter, HSM 5 a smaller lesser trochanter, HSM 6 a deeper superior curvature to the femoral neck and larger lesser trochanter, HSM 7 a smaller lesser trochanter and HSM 9 a smaller femoral head.

### HSMs 1 and 8 (pincer-type deformities) vs radiographic hip OA

In unadjusted analyses, HSM 1 was positively associated with the presence of RHOA, defined as Croft score ≥2 [OR 1.23 (1.09, 1.39)], whereas HSM 8 was negatively associated [OR 0.79 (0.70, 0.89)] (Table II). Results were unaffected by adjustment for age, weight, height and race. In terms of specific radiographic components, in adjusted analyses, HSM 1 was positively associated with the presence of acetabular [OR 1.13 (1.04, 1.22)] and inferior femoral [OR 1.22 (1.07, 1.38)] osteophytes (Table III), and with medial JSN [OR 1.55 (1.40, 1,71)] and subchondral sclerosis [OR 1.23 (1.09, 1.39)] (Table IV). Conversely, HSM 8 was inversely associated with osteophytes at all four locations (OR 0.78–0.89) (Table IV), and with lateral JSN [OR 0.71 (0.62, 0.82)] and subchondral sclerosis [OR 0.79 (0.69, 0.89)].

**Table II:** Associations of HSMs with the presence of RHOA based on the Croft score

	Unadjusted Croft ≥2		Adjusted Croft ≥2		Unadjusted Croft ≥3		Adjusted Croft ≥3	
	OR [95% CI]	*P*	OR [95% CI]	*P*	OR [95% CI]	*P*	OR [95% CI]	*P*
HSM 1	1.23 [1.09, 1.39]	0.00072*	1.23 [1.09, 1.39]	0.00082*	1.08 [0.89, 1.32]	0.43	1.10 [0.9, 1.35]	0.35
HSM 2	1.04 [0.92, 1.17]	0.56	1.01 [0.89, 1.14]	0.89	0.95 [0.78, 1.16]	0.63	0.93 [0.76, 1.14]	0.51
HSM 3	0.73 [0.65, 0.83]	3.6 × 10^−7^*	0.73 [0.65, 0.83]	4.0 × 10^−7^*	0.60 [0.50, 0.73]	3.7 × 10^−7^*	0.60 [0.50, 0.73]	3.5 × 10^−7^*
HSM 4	0.82 [0.73, 0.93]	0.0014*	0.83 [0.73, 0.93]	0.0021*	0.67 [0.55, 0.83]	0.00014*	0.69 [0.56, 0.84]	0.00028*
HSM 5	1.02 [0.91, 1.16]	0.71	1.03 [0.91, 1.17]	0.62	1.02 [0.83, 1.24]	0.88	1.01 [0.82, 1.24]	0.94
HSM 6	0.92 [0.82, 1.03]	0.14	0.92 [0.82, 1.03]	0.16	0.87 [0.72, 1.05]	0.15	0.86 [0.71, 1.05]	0.14
HSM 7	0.95 [0.84, 1.07]	0.40	0.98 [0.87, 1.11]	0.79	0.90 [0.74, 1.1]	0.30	0.96 [0.79, 1.18]	0.72
HSM 8	0.79 [0.70, 0.89]	0.00016*	0.78 [0.69, 0.88]	7.4 × 10^−5^*	0.64 [0.52, 0.79]	2.6 × 10^−5^*	0.63 [0.51, 0.78]	1.4 × 10^−5^*
HSM 9	0.95 [0.84, 1.07]	0.39	0.95 [0.84, 1.07]	0.41	0.93 [0.76, 1.14]	0.48	0.94 [0.77, 1.14]	0.52
HSM 10	1.21 [1.07, 1.37]	0.0020*	1.24 [1.1, 1.41]	0.00061*	1.29 [1.05, 1.59]	0.014	1.35 [1.1, 1.66]	0.0048*

Table shows results of logistic regression analysis between HSMs and Croft score in 4,100 individuals. Results show OR of having a Croft score per SD increase in HSM [95% confidence intervals] and *P*-value. Adjusted = adjusted analysis for age, weight, height and race. *P < 0.005.

**Table III:** Associations of HSMs with osteophytes at different sites

	Osteophyte Score	Lateral acetabulum		Lateral femoral		Inferior acetabulum		Inferior femoral	
		OR [95% CI]	*P* value	OR [95% CI]	*P* value	OR [95% CI]	*P* value	OR [95% CI]	*P* value
HSM 1	≥1	1.13 [1.04, 1.22]	0.0031*	1.11 [1.00, 1.24]	0.047	1.13 [1.02, 1.26]	0.019	1.22 [1.07, 1.38]	0.0022*
	≥2	1.01 [0.90, 1.14]	0.81	1.06 [0.91, 1.24]	0.45	1.18 [1.01, 1.39]	0.044	1.41 [1.10, 1.80]	0.0068
HSM 3	≥1	0.89 [0.82, 0.96]	0.0034*	0.78 [0.70, 0.87]	2.7 × 10^−6^*	0.76 [0.69, 0.85]	2.9 × 10^−7^*	0.80 [0.71, 0.90]	0.00035*
	≥2	0.79 [0.70, 0.89]	7.5 × 10^−5^*	0.60 [0.52, 0.71]	1.8 × 10^−10^*	0.73 [0.62, 0.86]	0.00017*	0.73 [0.57, 0.93]	0.011
HSM 4	≥1	0.92 [0.85, 1.00]	0.041	0.86 [0.78, 0.96]	0.0058	0.83 [0.75, 0.92]	0.00048*	0.86 [0.76, 0.97]	0.016
	≥2	0.96 [0.85, 1.08]	0.48	0.83 [0.71, 0.98]	0.024	0.83 [0.70, 0.97]	0.023	0.70 [0.55, 0.90]	0.0058
HSM 8	≥1	0.89 [0.82, 0.96]	0.0034*	0.81 [0.73, 0.90]	7.7 × 10^−5^*	0.84 [0.75, 0.93]	0.0011*	0.78 [0.69, 0.89]	0.00014*
	≥2	0.89 [0.79, 1.00]	0.046	0.74 [0.63, 0.87]	0.00025*	0.84 [0.71, 0.99]	0.035	0.77 [0.60, 0.99]	0.044
HSM 10	≥1	1.12 [1.03, 1.21]	0.0063	1.10 [0.99, 1.22]	0.082	1.12 [1.01, 1.25]	0.035	1.22 [1.07, 1.39]	0.0022*
	≥2	1.1 [0.98, 1.24]	0.11	1.17 [1.00, 1.37]	0.054	1.23 [1.04, 1.45]	0.017	1.40 [1.08, 1.82]	0.010

Table shows results of logistic regression analysis between HSMs and osteophytes, dependent on score ≥ 1 (any osteophyte) and ≥2 (moderate to severe osteophytes only), at different sites in 4,100 individuals. Results show OR of having any osteophyte per SD increase in HSM [95% confidence intervals] and *P* value, adjusted for age, weight, height and race. *P < 0.005.

**Table IV:** Associations of HSMs with JSN and subchondral sclerosis

	Lateral JSN		Medial JSN		Concentric JSN		Subchondral sclerosis	
	OR [95% CI]	*P* value	OR [95% CI]	*P* value	OR [95% CI]	*P* value	OR [95% CI]	*P* value
HSM 1	1.01 [0.87, 1.16]	0.91	1.55 [1.40, 1.71]	4.3 × 10^−17^*	1.23 [1.04, 1.45]	0.013	1.23 [1.09, 1.39]	0.0011*
HSM 3	0.73 [0.63, 0.84]	8.6 × 10^−6^*	1.32 [1.19, 1.46]	9.2 × 10^−8^*	0.98 [0.83, 1.15]	0.79	0.76 [0.67, 0.85]	6.8 × 10^−6^*
HSM 4	0.84 [0.73, 0.97]	0.020	1.32 [1.20, 1.46]	5.9 × 10^−8^*	1.05 [0.89, 1.25]	0.54	0.84 [0.74, 0.95]	0.0067
HSM 8	0.71 [0.62, 0.82]	4.3 × 10^−6^*	0.87 [0.78, 0.96]	0.0057	0.87 [0.74, 1.03]	0.11	0.79 [0.69, 0.89]	0.00018*
HSM 10	1.16 [1.01, 1.35]	0.039	1.11 [1.00, 1.23]	0.041	0.93 [0.79, 1.10]	0.39	1.21 [1.07, 1.38]	0.0025*

Table shows results of logistic regression analysis between HSM and JSN and subchondral sclerosis in 4,100 individuals. Results show OR of having any JSN or subchondral sclerosis per SD increase in HSM [95% confidence intervals] and *P* value, adjusted analysis for age, weight, height and race. *P < 0.005.

### HSMs 3, 4 and 10 (cam-type deformities) vs radiographic hip OA

In unadjusted analyses, HSM 3 and HSM 4 were inversely associated with prevalent RHOA [OR 0.73 (0.65, 0.83) and 0.82 (0.73, 0.93), respectively], whereas HSM 10 was positively related [OR 1.21 (1.07, 1.37)] (Table II). Equivalent results were seen in analyses adjusted for age, weight, height and race. In terms of specific radiographic components, in adjusted analyses HSM 3 was inversely related to the presence of osteophytes at all sites (OR 0.76–0.89) (Table III), and to lateral JSN [0.73 (0.63, 0.84)] and subchondral sclerosis [OR 0.76 (0.67, 0.85)], whereas there was a positive association with medial JSN [OR 1.32 (1.19, 1.46)] (Table IV). HSM 4 was inversely related to the presence of inferior acetabular osteophytes [OR 0.83 (0.75, 0.92)], but positively related to medial JSN [OR 1.32 (1.20, 1.46)]. HSM 10 was positively associated with inferior femoral osteophytes [OR 1.22 (1.07, 1.39)] and subchondral sclerosis [OR 1.21 (1.07, 1.38)]. Equivalent observations were seen in unadjusted analyses (data not shown).

### Association of hip shape with hip pain

In adjusted analyses, HSM 3 was inversely associated with hip pain on internal rotation [OR 0.84 (0.76, 0.92)] and on walking [OR 0.88 (0.81, 0.95)], and with WOMAC pain score [OR 0.87 (0.80, 0.93)]. Similar results were seen in unadjusted analyses (data not shown). There was weak evidence that HSM 4 was inversely associated with hip pain on examination and on walking and with WOMAC score, and that HSM 8 was positively related to these parameters (all *P* < 0.02); however, for all these *P* values were >0.005.

### Sensitivity analyses

Similar point estimates were observed for associations between HSMs and RHOA using a definition of Croft score ≥3 as opposed to ≥2 (Table II). Equivalent results were also obtained for associations between hip shape and moderate or severe osteophytes (i.e., grade ≥2), compared to those seen for grade ≥1 osteophytes as presented in the main results (Table III).

## Discussion

We examined associations between hip shape, as assessed by SSM performed on hip DXA scans, and prevalent RHOA ascertained approximately 5 years later, in a large population based cohort of older men. We found that five out of the top ten HSMs were associated with prevalent RHOA, and one mode was also associated with hip pain. Taken together, these findings suggest that SSM applied to hip DXA scans can be successfully used to identify shape changes associated with hip OA, particularly radiographic features. Given the substantial number of large population based cohorts with available hip DXA scans, this finding opens up the possibility of identifying novel genetic risk factors for hip OA, based on GWAS studies of DXA-derived hip shape.

All five HSMs associated with RHOA showed features of FAI, reflecting either cam- or pincer-type deformities. Given the cross-sectional nature of this analysis, it was not possible to distinguish shape changes resulting from hip OA, from those causing it. That said, our finding that three HSMs, indicative of cam-type deformity, are related to RHOA is consistent with previous studies suggesting that cam-type deformity is a risk factor for RHOA, based on SSM^14^, and measured geometric parameters^26^, ^27^. As well as being related to global RHOA as reflected by Croft score, HSM 3, HSM 4, and HSM 10 showed equivalent relationships with osteophytes at different sites, and in the case of HSM 3 with lateral JSN and subchondral sclerosis. HSM 3 also showed the strongest association with hip pain. However, the associations between hip shape and hip pain were generally weaker than that for RHOA. This lack of concordance between radiographic and clinical features of hip OA is well recognized^15^, and was supported by further analyses in which we examined associations between RHOA as defined by Croft score ≥2 and clinical features. Whereas RHOA was positively associated with pain on examination and on walking, these associations were relatively modest (RR 1.67 and 1.51, respectively).

Our observation that two HSMs reflecting pincer-type deformity were positively associated with RHOA is also consistent with the view that pincer-type deformity contributes to FAI, which is in turn thought to be an important cause of hip OA^5^. That said, there is little evidence that pincer-type deformity is associated with RHOA in the general population. Indeed, in a recent study of 720 individuals from the CHECK study, pincer-type deformity, as measured on radiographs based on the centre-edge angle, was found to be protective against incident OA^2^. One possible explanation for these apparently discrepant findings is that the relationship between pincer-type deformity and RHOA depends upon gender, since our present findings derived from the all-male MrOS cohort, whereas CHECK was 79% female.

Whilst the five HSMs associated with RHOA could be divided into those reflecting cam- and pincer-type deformities, these appearances may have arisen as a consequence of other OA related phenotypes. For example, the image resolution of DXA scans used in this study was too low to clearly visualize osteophytes, and so superior femoral osteophytes and lateral acetabular osteophytes may have been included inadvertently, leading to the impression of cam- and pincer-type deformities, respectively. Moreover, since we were only able to include the superior acetabulum in our SSM, we were unable to evaluate medial JSN and to exclude medial displacement of the femoral head as a cause of acetabular overhang, as opposed to pincer-type deformity. Our observation that HSM 1, which was positively related to pincer-type deformity, was also positively related to medial JSN on subsequent radiographs, is consistent with this alternative explanation.

As well as contributing to cam- and pincer-type deformities, HSMs may have reflected the presence of OA in other ways. For example, HSM 1 and HSM 3 were suggestive of greater supero-medial JSN, and HSM 8 lesser supero-lateral JSN. In addition, alteration in the size of the lesser trochanter associated with HSM 3 may reflect variation in the extent of internal rotation of the hip during image acquisition, which may in turn reflect underlying hip OA given the latter is associated with limited internal rotation. HSMs were also related to differences which may reflect other risk factors for developing OA apart from FAI. For example, HSM 1 was related to size of the femoral head and greater trochanter, and femoral neck width, which have recently been reported to be associated with prevalent radiographic knee OA^9^. That said, HSM 2 and HSM 9, which were also related to femoral head size but showed no relation to pincer- or cam-type deformities, were unrelated to RHOA or hip pain.

### Strengths and limitations

This study represents the first report of associations between DXA-derived hip shape and RHOA in a population based sample. The large size of the sample represents a further strength. The fact that this is a male cohort may have further increased power in light of previous findings suggesting the relationships between hip shape and RHOA are stronger in males than in females^28^, though to what extent our findings are applicable to females requires further study. Our study also highlights the benefit of using large DXA cohorts for hip shape research, though given the greater resolution of radiographs, the latter are more suitable in smaller studies, and in clinical practice. One limitation of this study was our lack of baseline radiographs. Therefore, the associations which we observed between HSMs, as assessed on baseline DXA scans, and RHOA based on radiographs collected 5 years later, could have reflected relationships with prevalent as opposed to incident OA. This distinction is important, since in examining associations with prevalent OA, it is difficult to infer causality, and the shape changes we observed could have been a result of, rather than a risk factor for, hip OA. A further limitation is the relatively low image resolution of the DXA scanner used in MrOS, making it difficult to determine to what extent associations between DXA-derived hip shape and RHOA reflected characteristics of established OA such as osteophytes, as opposed to shape changes representing possible OA risk factors such as those related to FAI. Newer DXA devices provide sufficient resolution for identifying osteophytes on hip DXA scans^29^. In addition, shape results could conceivably be affected by the degree of hip rotation; although the lower leg is strapped into a fixed position during scanning, the degree of hip rotation achieved might be affected by anatomical features such as pelvic size and shape, and associated hip disease. Another limitation, is that our HSMs cannot be directly applied to other cohorts since SSM using PCA is specific to the images used to make the model. Finally, SSM is not designed specifically to evaluate FAI and based on our findings more analysis, using methods specific to FAI such as alpha-angle, should be done to replicate these findings.

## Conclusions

Having applied a SSM of the femoral head and superior acetabulum to hip DXA scans from the MrOS cohort, we found that five out of the top ten HSMs were associated with RHOA, of which one mode was also related to hip pain. That these modes were associated with either cam- or pincer-type deformities is consistent with previous studies implicating FAI in the pathogenesis of hip OA. Furthermore, the observation that DXA-derived hip shape is related to prevalent hip OA suggests this may represent a useful phenotype for future GWAS studies intended to identify novel genetic risk factors for hip OA.

## Author contributions

BF, CG, RA, EO and JT conceptualized the study. BF, DB, JG, RB, JL, MN, NL and EO collected the data. BF, DB, CG, EO and JT developed the analysis plan and analysed the data. BF, RA, NL, EO, JT obtained funding. All authors contributed to the interpretation of the results, wrote the manuscript and have approved the final version of the manuscript. BF had full access to all the data and takes responsibility for its integrity and accuracy.

## Conflict of Interest

We have none to declare.
